# Bio-inspired melanin nanoparticles induce cancer cell death by iron adsorption

**DOI:** 10.1007/s10856-018-6190-x

**Published:** 2018-11-30

**Authors:** James Perring, Felicity Crawshay-Williams, Cindy Huang, Helen E Townley

**Affiliations:** 1 Department of Medical Sciences, Oxford University, Oxford, Oxfordshire UK; 2 Department of Women’s and Reproductive Health, Oxford University, Oxford, Oxfordshire UK; 3 Department of Engineering Science, Oxford University, Oxford, Oxfordshire UK

## Abstract

Dysregulation of iron metabolism is a common characteristic of cancer cells. The rapid proliferation of the tumour cells means that there is an increased dependence upon iron compared to healthy cells. Chelation of iron can be undertaken with a number of different compounds, however, simply lowering systemic iron levels to control tumour growth is not possible since iron is essential for cellular metabolism in the rest of the body. Nanoparticulate iron chelators could overcome this difficulty by targeting to the tumour either by the passive enhanced permeation and retention effect, or by targeting ligands on the surface. Nanoparticles were prepared from melanin, which is a naturally occurring pigment that is widely distributed within the body, but that can chelate iron. The prepared nanoparticles were shown to be ~220 nm, and could adsorb 16.45 mmoles iron/g melanin. The nanoparticles showed no affect on control fibroblast cells at a concentration of 200 μM, whereas the immortalised cancer cell lines showed at least 56% reduction in cell growth. At a concentration of 1 mM melanin nanoparticles the cell growth could be reduced by 99% compared to the control. The nanoparticles also show no significant haemotoxicity, even at concentration of 500 μM. Melanin nanoparticles are therefore a viable prospect for destroying cancer cells via iron starvation.

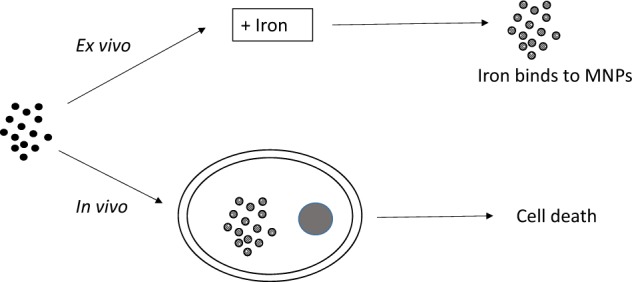

## Introduction

Iron is a trace element, integrally involved in a variety of metabolic processes from the synthesis of DNA to electron transport that underpins the production of ATP. These processes are upregulated in cells with a highly proliferative profile, such as cancer cells, meaning that acquiring sufficient amounts of iron is a crucial requirement if these cells are to survive. Cancer cells therefore exhibit an elevated dependence on iron when compared to healthy controls. To fuel this ‘iron addiction’, a range of metabolic alterations may occur that enhance the levels of cellular iron [1]. Such changes that abet neoplastic growth are therefore selected for within a tumour environment. As a result, dysregulation of iron metabolism is a common characteristic of malignant cell types, with increases in uptake and storage of iron, as well as reductions in its efflux, being frequently observed in these populations [2–6]. While it may seem that simply targeting and lowering systemic iron levels could control cancer growth, the essential role of iron in cellular metabolism throughout the body means that this is not a viable option. It is therefore necessary to develop a method of selectively targeting iron levels within tumour cells that has a minimal systemic activity. One approach is to use nanoparticles and to rely on the EPR (enhanced permeation and retention) effect, enabling the particles to passively accumulate within tumour cells, thus providing a simple method for producing selectivity of iron chelation [7].

A variety of iron chelation systems, many already in use in the clinic for treating diseases of iron overload, have been trialled for use in the treatment of cancer [8, 9]. However, most of these agents have short plasma half-lives and may elicit a host of adverse effects, such as hypersensitivity, neutropenia and GI complaints [10]. One of the most commonly used iron chelators is DFO. However, DFO is highly hydrophilic and has poor gastrointestinal absorption and a short half-life of approximately 12 min due to rapid metabolism [11]. As such, the compound is not orally active and needs to be administered by subcutaneous infusion for periods of 8–12 h from 5–7 times per week. The prolonged infusion can result in pain and swelling which results in poor patient compliance. Other iron chelators which have been explored for their potential to reduce cancer growth include Tachpyridine and Triapine. Tachpyridine has shown cytotoxicity against bladder cancer cells with an activity 15 times greater than that of DFO. Tachpyridine also binds Ca(II), Mg(II), Mn(II), Cu(II) and Zn(II) although it is thought that the cytotoxic effect is due to iron binding. Since tachpyridine arrests cells at G2, which is the radiosensitive phase of the cell cycle, it may also be used as a radiosensitizer [12]. This is in contrast to most iron chelators which arrest the cell cycle at the G1-S interface due to the inhibition of ribonucleotide reductase [13]. Triapine, whilst an effective chelator, is unlikely to be accepted for clinical medicine due to a number of serious side-effects including neutropenia, hypoxia, hypotension and methaemoglobinaemia [14].

The use of melanin, a pigment naturally occurring within the body that has been found to effectively chelate iron using in vivo mouse models, could therefore provide a more tolerable and effective alternative to the more commonplace pharmaceutical iron chelators [15]. In nature, melanins are widely distributed in many parts of the body and are involved in a range of functions ranging from photosensitisation, thermoregulation, protection from radiation and free radical quenching, as well as metal iron chelation. Within the body heavy metal ions such as iron and copper are tightly bound to melanin to protect cells from the Fenton reaction, and oxidative stress [16, 17].

To take advantage of the iron chelating characteristics of melanin and the targeting potential of the EPR effect, we prepared melanin nanoparticles and investigated their effects on immortalised cancer cell lines. In this study we tested the efficacy of the particles against two different rhabdomyosarcoma (RMS) lines and two different glioblastoma (GBM) lines. The RMS cell lines were each from a different histological subset; the RH30 cells being alveolar rhabdomyosarcoma (aRMS) cells and the RD cells embryonal rhabdomyosarcoma (eRMS) cells. eRMS make up 60% of all RMS cases and are characterised by small, round cells with a loose myxoid stroma, whereas aRMS are rarer, have a less favourable prognosis and are made up of discohesive round cells with interwoven fibrous septa [18, 19]. The GBM cell lines tested were U-87 MG and Mo59K. The U-87 MG line has a fast and aggressive growth in xenografts and a higher invasive potential than other GBM cell lines [20, 21]. The Mo59K cell line is slower growing than U-87 MG (Lu et al., in press [22]), and it has previously been reported that the sensitivity of different cell lines to apoptotic agents is related to their doubling time [23]. Both GBM cell lines are wild type for p53.

## Experimental

### Melanin nanoparticle synthesis

The synthesis of the water-soluble melanin nanoparticles (MNP’s) involves the hydroxylation of dopamine in alkaline solution. To synthesise the MNPs, 180 mg of dopamine hydrochloride (Sigma Aldrich) was dissolved in 90 ml of deionised water. While being stirred vigorously, 760 µL of 1 M NaOH solution was added to the dopamine hydrochloride solution at 50 °C. After ageing the mixture for approximately 5 h, the melanin nanoparticles were retrieved by centrifugation. First, the solution was centrifuged at low speed (4000 rpm) several times, each time being washed with deionised water to remove large-sized material. The required small nanoparticles were then obtained by high-speed centrifugation (18,000 rpm).

### Melanin nanoparticle characterisation

#### Zeta potential

The zeta potential of the melanin nanoparticles was measured using a Malvern Zetasizer Nano. Samples were prepared by dilution in ddH_2_0 (by drawing up a small amount of sample into a syringe already containing water), before being injected into a disposable zeta potential unit, being careful to ensure no air was trapped in the unit, before placing it into the zetasizer to begin measurement

#### Scanning electron microscopy

Samples were imaged using a Carl Zeiss Evo LS15 VP-scanning Electron Microscope. The specimens were first prepared by desiccating a sample of melanin nanoparticles before dissolving them in ethanol. The samples were then dry-casted on carbon adhesive discs and sputter-coated with 3 nm Au–Pd before imaging. The Scanning electron microscopy (SEM) images were secondary images taken with a primary energy of 15 kV.

#### Hydrodynamic diameter

The hydrodynamic diameter of the melanin nanoparticles was measured using a CPS disc centrifuge (DC 180000; CPS Instruments Europe). A sucrose density gradient was built up by injecting decreasing concentrations of sucrose into the centrifuge. The density gradient which is produced stabilises the particle sedimentation within the centrifuge. Samples (100 μl) were inserted into the centre of the disc and the time taken to reach the outside of the disc measured. The size distribution can then be calculated based on the density of the particles (assumed to be the bulk tabulated density) and the rotation speed. Samples were prepared by resuspending the melanin nanoparticles in ddH2O. Samples were calibrated against particles of a known diameter (Polyvinyl chloride, 0.377 µ, CPS Instruments Europe).

### Determination of melanin nanoparticle concentration

Having synthesised the water-soluble MNPs, the concentration was determined spectrophotometically. For each measurement, the spectrophotometer (Shimadzu UV-1800 Spectrophotometer) was first calibrated using a cuvette of ddH2O, before then inserting a cuvette of the desired solution and measuring the absorbance. From these measurements, the Beer–Lambert law could be used to calculate the concentration of the original stock solution. The Beer–Lambert law states that the absorbance (*A*) is equal to the molar extinction coefficient (*ε*) multiplied by the particle concentration (*c*) multiplied by the pathlength (*L*); *A* = *ε x c x L*. The wavelength used for this determination was 240 nm, and a molar extinction coefficient (*e* = 5953.2) was obtained from the literature [24].

### Cell culture

Two human Rhabdomyosarcoma (RMS) tumour cell lines (RH30, RD) were used for experimentation: RH30 (American Type Tissue Culture Collection [ATCC] no. CRL-20610, is an alveolar rhabdomyosarcoma (ARMS) and RD (ATCC no. CRL-7763) is an embryonal RMS (ERMS). A fibroblast control cell line (Fibroblasts) was also used (A kind gift from Dr J. Poulton). Cells were grown in Dulbecco’s Modified Eagles Medium - high glucose (DMEM; Aldrich) supplemented with 10% Foetal calf serum (FCS; Aldrich), 2 mM l-Glutamine (Aldrich), 100 U/ml Penicillin (Aldrich) and 0.1 mg ml^−1^ Streptomycin (Aldrich). Cells were incubated at 37 °C in a 5% CO_2_ atmosphere and passaged when confluent (approximately every 4 days).

### Cell count

Cells were seeded at a density of 1 × 10^4^ cells per well in 96-well plate. Cells were incubated at 37 °C in 5% CO2 atmosphere overnight to allow adherence to the plate. Melanin nanoparticles were then added to the media at concentrations of 0, 10, 50, 100, 200, 500 and 1000 μM. The cells were then returned to the incubator for 24 h. To assess cell viability after treatment, the cells were washed with 100 μL of PBS to remove nonadherent dead cells. Adherent live cells were then detached using 50 μL Trypsin-EDTA (Sigma-Aldrich). The enzyme activity was then neutralised with 50 μL of growth medium per well. The viable cells were then counted manually with a haemocytometer. These experiments were performed in triplicate on two separate occasions.

### Determination of cell Iron content

Cell lysate was prepared from cells which had been collected and washed as per Section 2.4 and resuspended in PBS. Cell suspension was then heated to 95 °C for 15 min, followed by sonication for 2 min using a VibraCell probe. Cell debris was removed by centrifugation at 12,000 rpm for 5 min, and the supernatant retained. Iron content was determined using the method of Ali et al. [25]. Briefly, into each well of a 96-well plate, 80 μl of 2M calcein, 20 μl of 500 mM HEPES pH7.4, and 20 μl of distilled water was added. To each well, either 80 μl of sample, or iron standard was added, in quadruplet repeats. The plate placed into a box with a lid, lined with moist tissue paper before incubating for 20 min at 37 °C. Subsequently, the absorbance was measured at Ex. 485 nm/ Em. 515 nm. Next, 5 μl of 1 mM DFO was added to two of the repeats, and 5 μl of water added to the two remaining repeats. The plate was incubated as before at 37 °C for 10 min and the absorbance of the samples measured at the same wavelengths.

### Protein determination

Protein quantification was performed using the Bradford’s assay. Bio-Rad protein assay dye reagent concentrate was diluted 1:5 as per manufacturer’s instructions. Bovine serum albumin was used as a protein standard to create a calibration curve. Absorbance was measured at 595 nm.

### Determination of iron binding by melanin

Melanin nanoparticles were prepared at stock solutions of 2 mM and 10 mM. Iron (II) sulphate heptahydrate (Sigma, Poole) stock solutions were prepared at double the final concentration required. Equal volumes of melanin nanoparticle and iron sulphate solution were mixed to give final concentrations of 1 mM and 5 mM melanin, in varying concentrations of iron. The solutions were incubated at 37 °C for either 24 or 48 h. After incubation the solutions were centrifuged at 15,000 rpm for 3 min and the supernatant containing unbound iron was collected.

To the collected supernatant (810 μl) add 1/10 volume of sodium acetate buffer (equal volumes of 6 M acetic acid and 5 M NaOH) and incubate at room temperature for 10 min. Next, add 10 μl of hydroxylamine solution (10 wt% in water), and incubate for a further 10 min. Phenathroline solution (prepared by adding 0.1 wt% phenathroline to heated water (60 °C) was then added, to give a final volume of 1 mL per sample. Sulphuric acid (8 μl) was added to each sample to ensure that the pH was in the range 4–7. The solutions were mixed and incubated for 20 min to allow the colour reaction to take place. The absorbance of the solutions was measured at 450 nm.

A calibration curve was obtained using a series of iron sulphate solutions (0, 0.32, 0.48, 0.8, 0.96, 1.62, 2.43, 3.25, 4.0, 6.8, 8.0 mM). A linear relationship was found (*R*^2^ = 0.9934).

### Blood interaction studies

Defibrinated horse blood was used to assess the haemotoxicity of the melanin nanoparticles. Blood (10 mL) was transferred to a centrifuge tube, and centrifuged at 2000 g for 5 min to pellet the red blood cells. The pellet was resuspended in PBS and centrifuged for 2000 g for 5 min. The wash was repeated twice more. After the final wash the supernatant was removed and the pellet resuspended in 1:10 (v/v) PBS. The sample was then divided into equal volumes in separate tubes. A positive control comprised adding 400 μL water to the sample, and a negative control used the same volume of PBS. The MNPs were added at different concentrations in 400 μL PBS. All samples were incubated at 37 °C for an hour. The samples were then centrifuged at 10,000 g for 5 min. Supernatant (100 μL) was taken from each tube and placed in a 96-well plate. The absorbance was measured at 595 nm. The experiment was repeated on three separate occasions.

### Prussian blue staining

Prussian blue staining was used to assess the degree of iron chelation in cells. Cells were seeded in 96-well plates at 1 × 10^4^ cells per well in 200 μl growth media and incubated overnight for cells to adhere. After the melanin nanoparticles were applied, the cells were left for another 24 h in the incubator. After this time, the media was removed by aspiration and the cells were washed with PBS. Cells were fixed in 100 μl of 1% (v/v) glutaraldehyde (aq; Sigma Aldrich) for 40 min. Perl’s reagent was freshly prepared by mixing equal parts of a 4% potassium ferrocyanide solution (aq; Sigma Aldrich) and 12% HCl (diluted from a 37% stock; Fisher Scientific). The glutaraldehyde was then removed and the cells were washed with PBS. The cells were incubated with Perl’s reagent for 10 min, before the stain was removed and fresh stain was re-applied before incubating for another 10 min. Following this staining process, the cells were washed with PBS three times. The cells were then counterstained using a 0.02% Neutral Red solution for 10 min before removing the stain and washing twice with distilled water. The stained cells were then observed and imaged using an inverted optical microscope

(Motic AE31).

### Statistical analysis

Significance was tested using a two-tailed equal variance *T*-test comparing treated and untreated cells (*p ≤ 0.05, **p ≤ 0.01, ***p ≤ 0.005).

## Results

### Iron levels in immortalised cancer cell lines

**Fig. 1:**
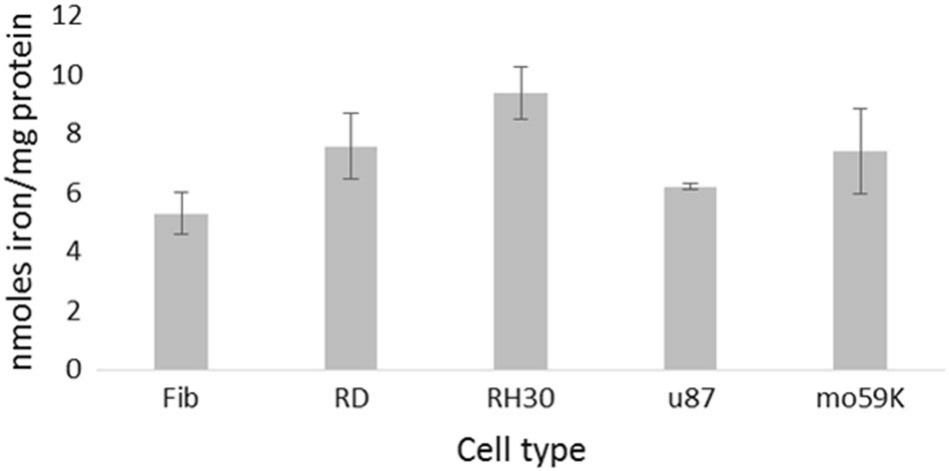
Iron levels in RMS and GBM cell lines. Cells from the different origins were homogenised and total iron measured relative to the protein concentration. Data shown represents *n* = 3 sample repeats, and *n* = 3 independent experiments. Data is shown as average ± standard deviation

The levels of free iron present in the cytosol of immortalised cancer cell lines was assessed using a colourimetric assay (Fig. 1). A control fibroblast line was shown to have the lowest iron content of 5.27 ± 0.71 nmol/mg. There was a significant increase in iron content in RD cells (7.57 ± 1.10 nmol/ mg; *p* < 0.05) and RH30 cells (9.36 ± 0.90 nmol/mg; *p* < 0.005). Interestingly RH30 cells have been shown to be more metastatic than RD cells, and iron is needed for cells with high proliferation rates. There was also an increased amount of iron in U-87 MG cells (6.09 ± 0.09 nmol/mg) and Mo59K cells (7.37 ± 1.45 nmol/mg), although this was not statistically significant compared to fibroblasts. These values for the iron content of the cells are consistent with those from the literature, where for example the basal total iron content of confluent astrocyte cultures was found to be 10.2 ± 2.9 nmol iron per mg protein [26].

### The iron chelator DFO is not an effective chemotherapeutic

**Fig. 2:**
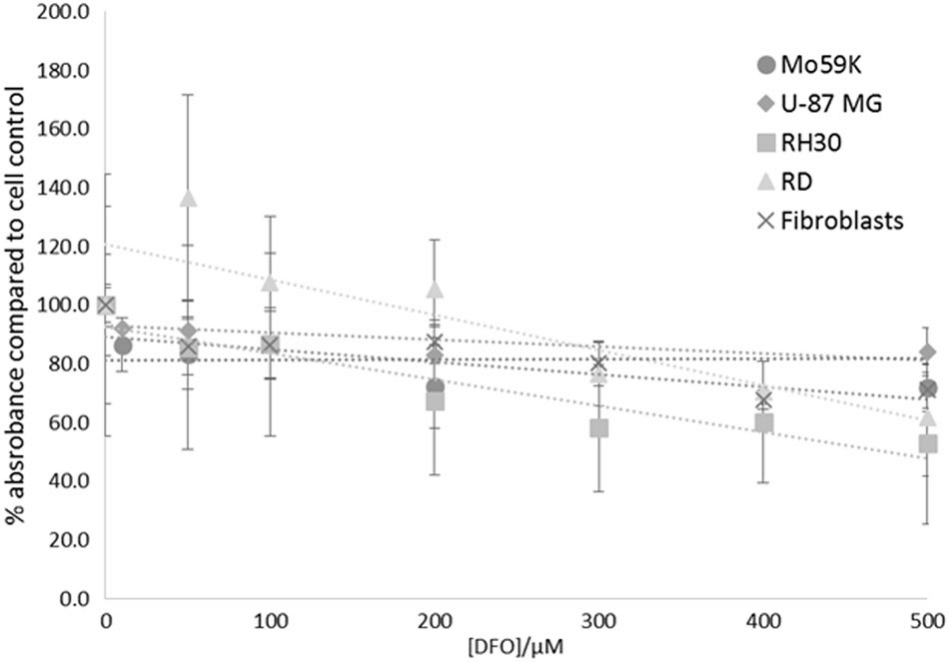
Effects of varying concentrations of desferrioxamine (DFO) on cell survival. Cell lines were incubated with DFO for 24 h to assess the effect on cell survival. (●) Fibroblast cells (■) RH30 cells (▲) RD cells (♦) U-87 MG (x) Fibroblasts. Data shown represents *n* = 3 sample repeats, and *n* = 2 independent experiments. Data is shown as average ± standard deviation

DFO is an intracellular iron chelator, and is used in clinical practice for removing excess iron in patients with conditions such as haemochromatosis. We therefore investigated the effect of DFO as an iron chelator with the potential to cause selective cell death in cancer cells. Our results showed that there was a linear decrease in cell number for all three cell lines in the presence of up to 500 μM DFO (Fig. 2). Cells were also tested at concentrations up to 4000 μM but no further decrease in cell number was observed (data not shown). After incubation with 500 μM DFO, fibroblasts showed 71.1% ( ± 6.15) cell survival which showed no statistically significant difference to the zero control (*p* = 0.1576). RD cells showed greater cell death with 61.8% ( ± 19.99) cell survival with 500 μM DFO, but this also showed no statistically significant difference to the zero control (*p* = 0.2261). RH30 cells, however, showed 52.9% (±27.31) cell survival, and were significantly different from the zero control (*p* = 0.01693). The brain cancer lines were the least affected by DFO and showed 71.9% ± 8.0 (U-87 MG) and 84.1 % ± 8.0 (Mo59K) cell survival after incubation with 500 μM DFO, although these were both significantly different from the control (*p* < 0.005). Overall, DFO showed no more than 50% cell death over 24 h in any of the cell lines, and fibroblasts were affected to a similar degree to the cancerous cells, and so there was no selectivity. This means that DFO is by no means an ideal compound to use for cancer cell death.

### Melanin nanoparticles induce cell death only in cancerous cells

#### Synthesis of melanin nanoparticles

**Fig. 3:**
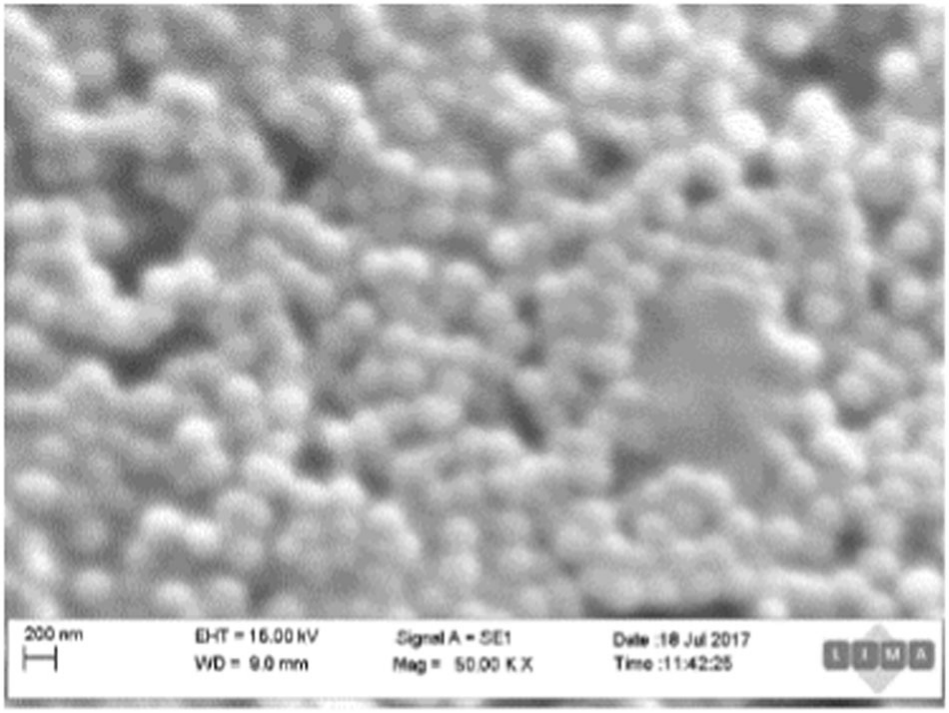
Scanning Electron Microscope image of melanin nanoparticles. The image shows spherical melanin nanoparticles. The scale bar is 200 nm

The hydrodynamic diameter, and size distribution of the nanoparticles was determined using a CPS disc centrifuge. The mean particle diameter was calculated to be 220.5 ± 51.34 nm, with a mean polydispersity index of 1.21 ± 0.023. The melanin nanoparticles were imaged using scanning electron microscopy to determine the morphology of the nanoparticles. SEM images (Fig. 3) showed the particles to be roughly spherical. The diameter is estimated to be between 200–300 nm, which is consistent with the hydrodynamic size distribution obtained from the disc centrifuge testing of the samples. In addition to the size, the charge of the nanoparticles will determine how they will interact with cells. The Zeta Potential of the particles was therefore assessed, and determined to be −24.4 ± 0.771 mV.

#### Melanin adsorption of iron

**Fig. 4:**
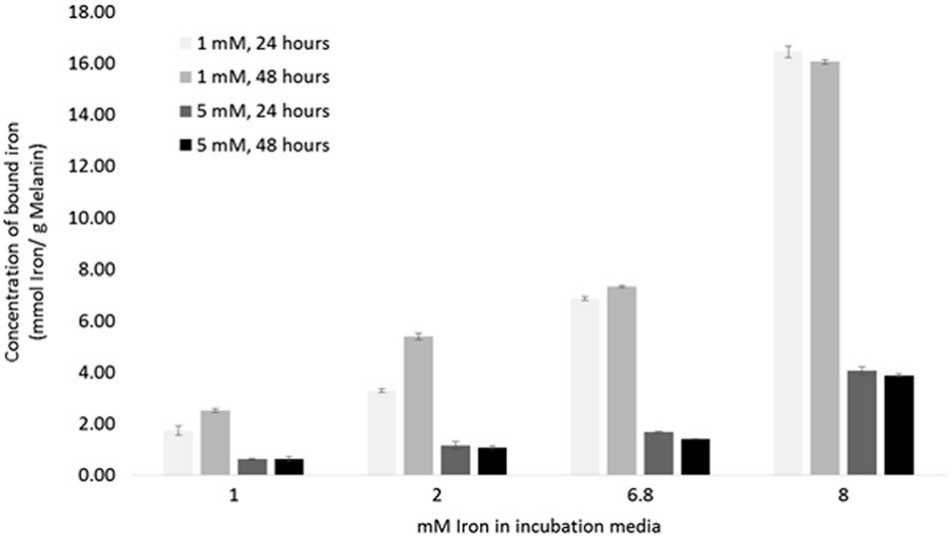
Melanin nanoparticle Iron chelation. Melanin nanoparticles (1 mM and 5 mM) were incubated with an iron sulphate solution (1, 2, 6.8 and 8 mM). The particles were incubated in the iron solutions for either 24 or 48 h. Data shown represents *n* = 3 sample repeats, and *n* = 2 independent repeats. Data shown is average ±standard deviation

Once the melanin nanoparticles had been characterised in terms of their physical properties, they were assessed for their ability to bind iron. The nanoparticles were incubated in solutions of increasing concentrations of iron, and were tested for two different concentrations of nanoparticles, and for two different incubation periods (Fig. 4). It can be seen that a solution of 1 mM melanin particles was able to adsorb the most iron per gram of material, and that there was no significant increase when the particles were incubated for 48 h rather than 24 h. This indicates that the particles are unable to adsorb any more iron from the solution. The highest adsorption in our system was 16.45 ± 0.21 mmoles iron/ g melanin. The amount of iron adsorbed per gram of material was lower when a 5 mM particle solution was prepared, for both 24 and 48 h. This suggests that at a higher concentration the nanoparticles had not reached their loading capacity.

#### MNPs induce cell death only in cancerous cells

**Fig. 5:**
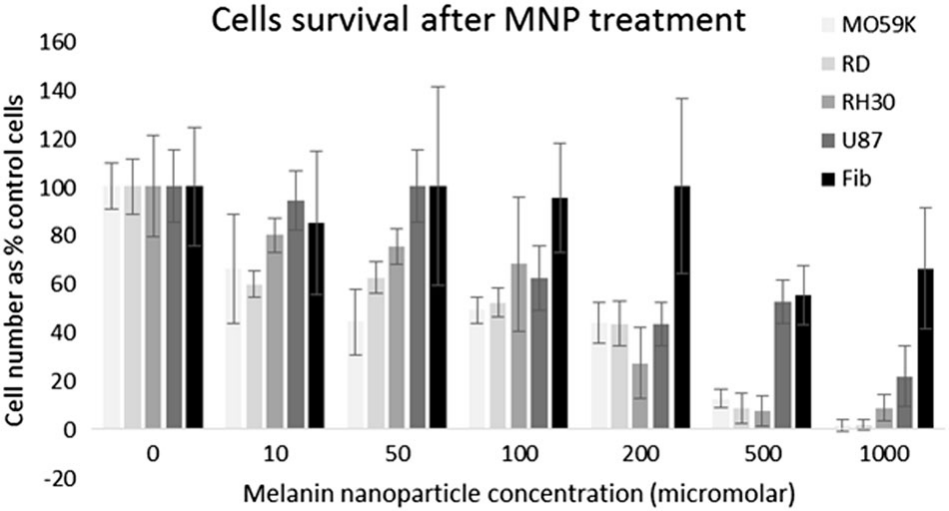
Cell survival after iron chelation. Cell lines were incubated with increasing concentrations of melanin nanoparticles, for 24 h. Data shown represents *n* = 3 sample repeats, and *n* = 3 independent repeats. Data shown is average ± standard deviation

Control fibroblast cells were tested with a range of the melanin nanoparticles, and up to 200 μM nanoparticles there was no significant difference in cell death from the zero control. After incubation with 500 μM and 1000 μM nanoparticles, cell viability was significantly reduced to 55.0% ± 12.24 (*p* < 0.005) and 66.0% ± 25.1 (*p* < 0.05), respectively (Fig. 5). At a concentration of 200 μM nanoparticles all of the cancer-derived cell lines show significantly greater cell death than control fibroblast cells. This same trend is seen at 500 and 100 μM nanoparticles (with the exception of U-87 MG cells at 500 μM nanoparticles). While there is no direct evidence that iron chelation is responsible for cell death, we have shown that the melanin nanoparticles can effectively chelate iron (Fig. 3) and that they increase cell death preferentially in cancerous cells (Fig. 5).

#### Iron staining in cells incubated with melanin nanoparticles

**Fig. 6:**
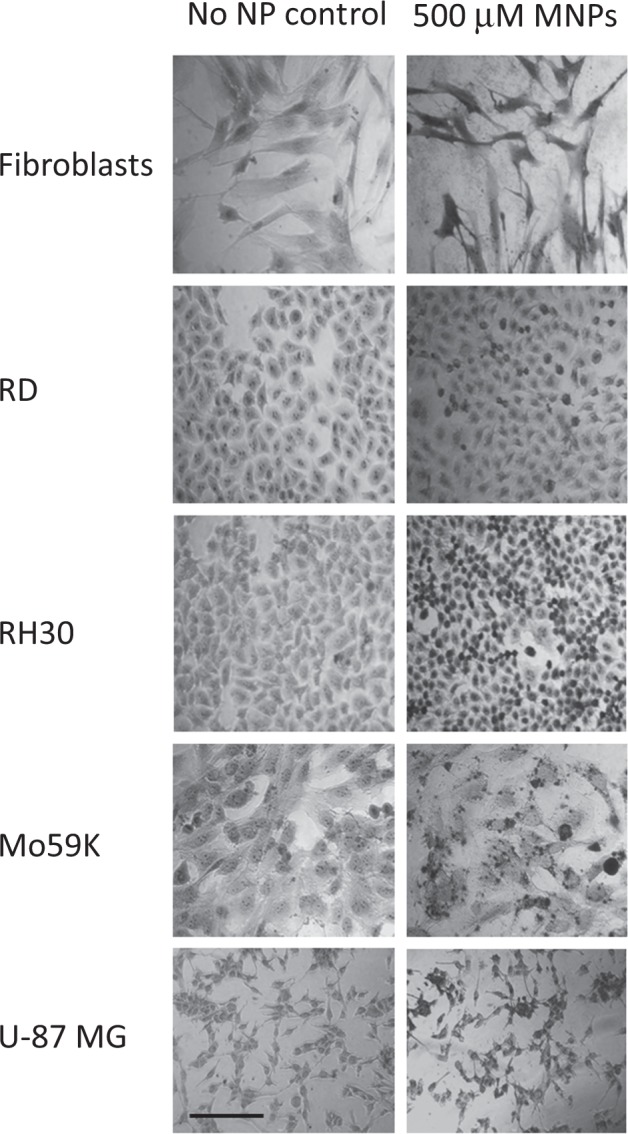
Iron staining of immortalised cells. Cells were incubated with melanin nanoparticles for 24 h and compared to controls. The cells were fixed and stained with Perl’s reagent and Neutral Red. Representative images are shown. Scale bar shows 100 µm

To both determine the localisation of the nanoparticles within the cells and to indicate the level of iron chelation mediated by the melanin system, the cells were stained with Perls’ reagent. The Perls’ reagent forms an insoluble complex with iron, staining it an intense shade of blue and allowing areas that are particularly rich in iron (e.g. melanin nanoparticles that have chelated iron from within cells) to be clearly visualised [27]. The neutral red is used as a counter-stain to provide contrast to the blue iron-complexes (Fig. 6). The nanoparticles appeared as dark-stained regions confirming the results shown in Fig. 4 that they were effective in their ability to chelate iron.

The images indicate that the nanoparticles were able to enter the cancer cells; this is more likely than the particles remaining on cell surfaces since chelation of intracytoplasmic iron and chelation-induced death would require cell internalisation. The reduced volumes of the cells seen in the images also indicates that cell death is occurring.

#### Melanin nanoparticles do not induce haemotoxicity

**Fig. 7:**
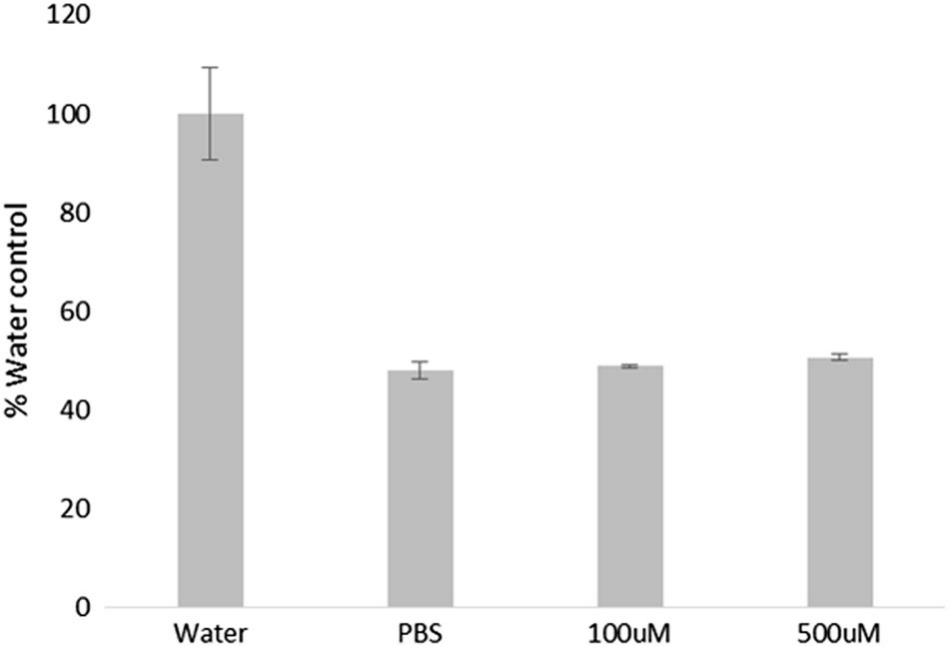
Haemotoxicity assay of melanin nanoparticles. Blood was incubated with either water, PBS, or melanin nanoparticles, for 24 h. The serum was analysed spectroscopically to detect whether blood cell lysis had occurred. Data shown represents *n* = 3 sample repeats, and *n* = 3 independent repeats. Data shown is average ± standard deviation

The melanin nanoparticles were tested for their interactions with red blood cells (Fig. 7). The degree of haemolysis was measured against that caused by adding water. The negative control, PBS alone, was shown to result in 48.01% (±1.74) of the cell lysis with water. There was no significant difference (*p*=0.35) between PBS alone and 100 μM melanin nanoparticles (48.94%±0.367). A further, higher, concentration was tested (500 μM) for which very marginally higher (50.74% ± 0.530) lysis was observed.

## Discussion

The results from this study indicate that nanoparticulate melanin, likely acting through iron adsorption, is able to induce significant cell death in tumour cell lines, with selectivity for malignant cell lines over control fibroblast cells.

Tumours are iron consumers; the element facilitates rapid proliferation and growth, and is an important contributor to tumour angiogenesis. Breast cancer cells have been shown to have abnormal pathways for iron acquisition, storage and regulation, which suggests that the reprogramming of iron metabolism is an important aspect of cancer cell survival. Furthermore, the iron and the microenvironment of the breast cancer cells may protect them from being destroyed by natural killer cells [28]. A number of iron chelating agents have shown potential for the treatment of malignancy, such as Deferasirox, which was able to induce complete remission in a previously chemotherapy-resistant Acute Monocytic Leukaemia in one patient [29]. DFO which is classically used to treat haemochromatosis, or acute iron poisoning has also been trialled for cancer treatment. Cancer stem cells, for example, have been shown to suppress the proliferation of miPS-LLCcm cells and the expression of stemness markers [30]. However, the compound is difficult to produce and therefore expensive, and requires frequent doses due to its short half-life. Furthermore, DFO has been shown to increase metastasis in colorectal cancer, most likely through its status as a hypoxia mimetic and increased expression of HIF-1α [31].

Melanin is a biological pigment which is known to chelate various metal ions, such as Fe^3+^, Cu^2+^, Mg^2+^, Ca^2+^, Zn^2+^, Pb^2+^, either via hydroxyl groups or via amine groups [15]. Melanin nanoparticles therefore present an ideal prospect for chelating iron at the tumour site. The possibility for localisation mitigates the risk of depriving the rest of the body of iron while still depleting supplies to the cancerous cells. We have shown that the melanin nanoparticles can bind 16.45 ± 0.21 mmoles iron/g melanin (Fig. 3), and if we assume an approximate value of 10 nmoles iron/mg protein in cancerous cells (Fig. 6); for a 1 cm tumour containing ~1 × 10^9^ cells with an average of 300 pg protein/cell it would take 0.18 mg of the melanin nanoparticles to bind the iron in the cells [32, 33]. Whilst this is of course a very rough approximation it shows that the quantity of nanoparticles required is reasonable, and a feasible amount to be taken up by a tumour.

The melanin nanoparticles were shown to be able to induce cell death specifically in cancer cells. At a concentration of 1000 μM melanin nanoparticles, 66.00% ± 25.1 of fibroblast cells survived, compared to the cancerous cell lines where survival was much lower (Mo59K cells: 1.01% ± 23.55; RD cells: 1.35±2.26; RH30 cells: 8.33±5.38; U87-MG cells: 21.4±12.5). The data (Fig. 5) shows that while the cancer cells are all more sensitive than the control fibroblast cells, there is still variation in sensitivity between the cancerous cell lines. This is in contrast to the commonly used metal chelator, DFO, which showed no more than 50% cell death over 24 h. Therefore melanin nanoparticles can be seen to be a much more effective agent for causing cell death, most likely by removing iron (although other mechanisms cannot be excluded at present).

The haemolytic potential of all intravenously administered pharmaceuticals needs to be evaluated since haemolysis, which results from damage to red blood cells (RBCs) can lead to anaemia, jaundice and other pathological conditions [34]. In particular, the unique physicochemical properties of nanoparticles may cause their interactions with red blood cells to differ from those of the bulk material, or conventional pharmaceuticals. Particle-induced haemolysis was measured by determining the amount of haemolysis determined spectroscopically by detecting free haemoglobin after centrifugal separation from the nanoparticles, and undamaged red blood cells. The melanin nanoparticles did not show significant haemolysis. This is as expected since melanin is a biopolymer known to have good biocompatibility [15].

## Conclusions

The chelation of iron from tumours can be effective in preventing growth and proliferation due to the reliance of rapidly growing cells on iron for replication. A number of molecules are available for the chelation of iron in vivo but these suffer from lack of efficacy and a number of side effects. The utilisation of the natural molecule melanin, in nanoparticulate form, has been shown to adsorb iron and to effectively cause cancer cell death while showing minimal cell death in normal fibroblast cells. Thus, melanin nanoparticles could be expected to be an effective cancer treatment with minimal side effects.
